# Risk Factors for Macro- and Microvascular Complications among Older Adults with Diagnosed Type 2 Diabetes: Findings from The Irish Longitudinal Study on Ageing 

**DOI:** 10.1155/2016/5975903

**Published:** 2016-05-15

**Authors:** Marsha L. Tracey, Sheena M. McHugh, Anthony P. Fitzgerald, Claire M. Buckley, Ronan J. Canavan, Patricia M. Kearney

**Affiliations:** ^1^Department of Epidemiology and Public Health, University College Cork, Western Road, Western Gateway Building, Cork, Ireland; ^2^Department of Statistics, University College Cork, Western Road, Western Gateway Building, Cork, Ireland; ^3^Department of Public Health, Health Service Executive (HSE) South, St. Finbarr's Hospital, Douglas Road, Cork, Ireland; ^4^Department of Endocrinology, St. Vincent's University Hospital, Elm Park, Dublin 4, Ireland

## Abstract

*Objective*. To explore risk factors for macro- and microvascular complications in a nationally representative sample of adults aged 50 years and over with type 2 diabetes in Ireland.* Methods*. Data from the first wave of The Irish Longitudinal Study on Ageing (TILDA) (2009–2011) was used in cross-sectional analysis. The presence of doctor diagnosis of diabetes, risk factors, and macro- and microvascular complications were determined by self-report. Gender-specific differences in risk factor prevalence were assessed with the chi-squared test. Binomial regression analysis was conducted to explore independent associations between established risk factors and diabetes-related complications.* Results*. Among 8175 respondents, 655 were classified as having type 2 diabetes. Older age, being male, a history of smoking, a lower level of physical activity, and a diagnosis of high cholesterol were independent predictors of macrovascular complications. Diabetes diagnosis of 10 or more years, a history of smoking, and a diagnosis of hypertension were associated with an increased risk of microvascular complications. Older age, third-level education, and a high level of physical activity were protective factors (*p* < 0.05).* Conclusions*. Early intervention to target modifiable risk factors is urgently needed to reduce diabetes-related morbidity in the older population in Ireland.

## 1. Introduction

Over the past two decades, the global burden of diabetes has increased significantly [1–3]. Between 1998 and 2015, the prevalence of diabetes increased from 2.2% to 5.2% among the adult population in Ireland [4]. In 2010, diabetes was the ninth leading cause of mortality [1] and the 14th largest cause of disability adjusted life years (DALYs) [2] worldwide. The economic cost of diabetes is also high and will continue to rise; approximately 12% of the world's total health expenditure was spent on diabetes in 2010 [5]. The vast majority of this burden is attributable to the macrovascular (myocardial infarction, congestive cardiac failure, and cerebrovascular accident) and microvascular (diabetic neuropathy, retinopathy, and nephropathy) complications of diabetes [3]. The Cost of Diabetes in Europe-Type II study reported that 72% of people had at least one diabetes-related complication [6]. In Ireland, the prevalence of macro- and microvascular complications among adults aged 50 years and over with type 2 diabetes is 15% and 26%, respectively [7].

Compared to the general population, individuals with type 2 diabetes are at an increased risk of developing cardiovascular disease (CVD) [8, 9]. Type 2 diabetes is also a significant cause of blindness in adults, nontraumatic lower limb amputations, and end-stage renal disease resulting in transplantation and dialysis [3]. We reported that the risk of visual impairment, in adults aged 50–69 years, is approximately four times higher in the population with diabetes compared to those without diabetes [10]. Buckley et al. [11] found that the risk of an individual with diabetes undergoing lower-leg amputation was 22 times that of an individual without diabetes.

Time-related variables, such as age and longer diabetes duration, are associated with the onset of diabetes-related complications [12]. Established risk factors for CVD such as hypertension, high cholesterol, and smoking further increase the likelihood of developing both macro- and microvascular complications [13]. Socioeconomic status (SES) has also been identified as a predictor of microvascular complications. Lower educational attainment is considered to influence the development of such complications through health behaviours, access to care, and processes of diabetes care [14–17]. While the national prevalence of diagnosed type 2 diabetes and related complications has been established among the older population in Ireland [7], evidence on the individual level risk factors for macro- and microvascular complications is lacking. Identification and treatment of risk factors can delay or prevent the development of diabetes-related complications [3]. Therefore, the purpose of this paper is to identify the determinants of macro- and microvascular complications among adults aged 50 years and over with diagnosed type 2 diabetes.

## 2. Material and Methods

### 2.1. Data Source

Data from the first wave (2009–2011) of The Irish Longitudinal Study on Ageing (TILDA), a population-based prospective cohort study of community-dwelling adults aged 50 and over in Ireland, were used in this cross-sectional analysis [18]. A nationally representative sample was selected using the RANSAM sampling technique from a listing of all residential addresses in Ireland (the Irish Geodirectory) [19]. A total of 8175 adults aged 50 years and over completed a computer-assisted personal interview (CAPI), representing a household response rate of 62%. The CAPI was administered by trained social interviewers in the participants' homes. This recorded detailed information on health, social, and economic circumstances. During the CAPI, participants reported their medication use and interviewers noted the correct name from the medication packaging. Medications were assigned World Health Organization Anatomic Therapeutic Chemical (ATC) classification codes [20]. Ethical approval was obtained from the Faculty of Health Sciences Research Ethics Committee of Trinity College Dublin. Written informed consent was provided by all respondents before participation [18].

### 2.2. Type 2 Diabetes Classification

Methods to classify diagnosed type 2 diabetes and macro- and microvascular complications have been reported in detail elsewhere [7]. In brief, individuals were classified as having diagnosed diabetes if they self-reported a previous doctor diagnosis or if they reported the use of insulin or oral hypoglycaemic agents during the CAPI. Age at diabetes diagnosis (years) was established by self-report. Anyone aged less than 40 years at diagnosis and injecting insulin but not on oral hypoglycaemic agents was classified as having type 1 diabetes; all others were classified as having diagnosed type 2 diabetes [21].

### 2.3. Macrovascular and Microvascular Complications

All TILDA participants were asked the question “*Has a doctor ever told you that you have any of the conditions on this card?*” Heart attack (myocardial infarction), heart failure (congestive cardiac failure), stroke (cerebrovascular accident), and mini stroke (transient ischemic attack (TIA)) were included in the list and were defined as macrovascular complications. Participants who reported a doctor diagnosis of diabetes were asked the question “*Has a doctor ever told you that you have any of the following conditions related to your diabetes?*” The conditions listed were leg ulcer, protein in urine (proteinuria), lack of feeling and tingling pain in legs and feet due to nerve damage (diabetic neuropathy), damage to the back of your eye (diabetic retinopathy), or damage to your kidneys (diabetic nephropathy); these were defined as microvascular complications [21]. Macro- and microvascular complications were collapsed into dichotomous variables, to indicate the presence or absence of at least one complication.

### 2.4. Covariates

Sociodemographic, behavioural, and medical history variables were recorded in the CAPI including sex, age (50–64 years, 65–74 years, and 75+ years), location of household (capital city (Dublin), another town/city, and rural), educational attainment (primary-level or none, secondary-level, and third-level or higher), and medical care cover (means-tested public health insurance scheme for those on low incomes, supplementary private health insurance, dual cover (both state-assisted cover and private insurance), and no additional cover for healthcare). Smoking status was classified as having ever smoked (current and former smoker) and never smoked (nonsmoker). Physical activity (low, moderate, and high) was self-reported using the short version of the International Physical Activity Questionnaire (IPAQ) and categorised using the IPAQ scoring protocol [22]. Duration of diabetes diagnosis was calculated by subtracting age at diagnosis from age (years) at interview and was subsequently categorised (0–4 years, 5–9 years, and ≥10 years). The use of diabetes medication (oral hypoglycaemic agents, insulin, or none) was reclassified as diet alone, oral agents alone, insulin alone, or oral agents and insulin. A previous doctor diagnosis of hypertension or high cholesterol was ascertained by self-report.

### 2.5. Statistical Analysis

Analysis was carried out in Stata version 13 for Windows (StataCorp, College Station, TX) using the survey function (svy). Inverse probability weights were applied to all analyses to provide population estimates. Weights were calculated according to the distribution of marital status, educational attainment, and geographic location using Irish census data from the period of 2006–2010 and to the distribution of age and sex using Irish census data from 2011 [23].

Descriptive statistics were used to summarise characteristics of the population with diagnosed type 2 diabetes and were stratified by gender. Gender-specific differences in categorical variables were analysed using Pearson's chi-square test. The mean and standard deviation were reported if continuous data conformed to normality and Student's *t*-test was conducted to compare means. If data were skewed, the median with associated lower and upper quartile values was reported and the Mann-Whitney test was utilised.

Associations between risk factors and diabetes-related complications were examined using a log-binomial regression. Risk ratios (RR) and 95% CI were generated as a measure of association. Risk factors served as independent variables and were chosen on the basis of previous literature [12, 14, 24–35] or if significant associations were observed in univariate analysis. A forward blockwise entry method was used with independent variables which were entered into the regression model in three blocks: (1) sociodemographic variables (age, sex, and education attainment) and duration of diabetes diagnosis, (2) behavioural factors (ever smoking and level of physical activity), and (3) medical history variables (diagnosis of hypertension, high cholesterol). Collinearity was assessed by the variance inflation factor (VIF); a VIF of >10 indicated multicollinearity. Statistical significance was defined as *p* < 0.05.

## 3. Results

Of the 8175 participants in TILDA, 634 participants reported a previous doctor diagnosis of diabetes and 38 participants did not report a diagnosis of diabetes but were classified as having diabetes by the use of oral hypoglycaemic agents or insulin. Of these, 17 people were classified as having type 1 diabetes and were excluded from the present analysis. Of the 655 people with type 2 diabetes, 57.7% were male and the mean age was 66.6 (SD = 8.8) years.  Table 1 shows the characteristics of the population with type 2 diabetes, stratified by gender. Approximately half of the participants were diagnosed with diabetes between the ages of 50–69 years (51.8% [47.6% to 55.9%]). The median time since diabetes diagnosis was 5.3 years (IQR 2 to 11 years). Approximately half the study sample had completed secondary- or third-level education (51.1% [46.9% to 55.4%]). In terms of disease management, 17.5% (14.8% to 20.6%) of the participants managed type 2 diabetes with diet alone, 74.2% (70.6% to 77.5%) reported the use of oral hypoglycaemic agents, and 7.5% (5.6% to 10.0%) reported using both oral hypoglycaemic agents and insulin. The prevalence of cardiovascular risk factors was high among participants; 18.8% (15.8% to 22.3%) of the participants were current smokers, 62.5% (58.1% to 66.6%) reported a previous doctor diagnosis of hypertension, and 51.7% (47.5% to 55.9) reported a previous doctor diagnosis of high cholesterol. Current smoking was higher among females compared to males (20.6% [15.5% to 26.5%] versus 17.5% [13.9% to 26.5%]) and a higher proportion of males reported higher levels of physical activity (27.5% [23.2% to 32.3%] versus 13.7% [9.6% to 19.2%]). Fifteen percent (15.1% [12.3% to 18.4%]) of the participants reported a previous doctor diagnosis of at least one macrovascular complication and 26% (25.5% to 29.9%) of the participants reported a previous doctor diagnosis of at least one microvascular complication.

### 3.1. Factors Associated with Diabetes-Related Complications

Table 2 presents the results from the binomial regression analyses, where previous diagnosis of at least one macrovascular complication served as the dependant variable. The risk of a macrovascular complication was higher among older participants (RR 1.6 [1.1 to 2.5] in 65 to 74 years; RR 2.0 [1.2 to 3.2] in 75 years or over versus 50 to 64 years). Female participants were less likely to report a previous diagnosis of a macrovascular complication relative to males (RR 0.6 [0.4–0.8]). Finally, individuals classified as having ever smoked had a 60% increase in the risk of a macrovascular complication compared to those who never smoked (RR 1.6 [1.1 to 2.6]).

Table 3 presents the results from the binomial regression analyses, with previous diagnosis of at least one microvascular complication as the dependant variable. There was no evidence to suggest that the risk of microvascular complications was different in participants diagnosed for less than four years compared to those diagnosed for a period between five and nine years (RR 1.1 [0.8 to 1.7]), whereas participants with type 2 diabetes for 10 or more years were approximately twice as likely to have reported a microvascular complication compared to those who had been diagnosed for less than four years (RR 1.9 [1.4 to 2.5]). Participants who had ever smoked were almost one and a half times more likely to report a doctor diagnosis of a microvascular complication relative to those who never smoked (RR 1.4 [1.1 to 2.0]). While the risk of a microvascular complication did not differ between those with a secondary-level education compared to those with primary-level or less education (RR 0.9 [0.6 to 1.2]), the risk was significantly decreased in participants with a third-level education (RR 0.6 [0.4 to 0.9]) compared to those with primary-level or less education. The risk of a microvascular complication did not differ between participants who reported a moderate level of physical activity compared to those with low level of activity (RR 0.8 [0.6 to 1.1]). However, participants from the highest physical activity category were 50% less likely to report a microvascular complication (RR 0.5 [0.3–0.8]) compared to participants with low level of activity. Participants diagnosed with hypertension were one and a half times more likely to have reported a previous diagnosis of at least one microvascular complication relative to participants who had not reported a previous diagnosis (RR 1.5 [1.1–2.1]). Collinearity was not found between variables (VIF ≤ 2).

## 4. Discussion

To our knowledge, this is the first study to identify individual level risk factors associated with macrovascular and microvascular complications among people with type 2 diabetes in Ireland using nationally representative data. Older age, having ever smoked, and a previous doctor diagnosis of high cholesterol were independently associated with an increased risk of macrovascular complications, while being female and high levels of physical activity demonstrated a protective effect. Longer duration since diagnosis, having ever smoked, and a previous doctor diagnosis of hypertension were associated with an increased risk of microvascular complications whereas achieving a third-level or higher education and high levels of physical activity demonstrated a protective effect.

Consistent with existing research [28, 29], established risk factors for CVD [28, 29] and being male [28, 36] were found to be independent predictors of at least one macrovascular complication. The United Kingdom Prospective Diabetes Study (UKPDS) reported that the risk of myocardial infarction and stroke were higher in older participants, smokers, and those with high cholesterol [28, 29]. Prospective studies have also identified hypertension [28–30, 32] as a risk factor for the development of macrovascular complications among individuals with type 2 diabetes; however, our study failed to demonstrate a significant association. Our findings are not atypical of existing research in this area; the diversity of results has been discussed previously [28–30, 37]. Evidence demonstrating an association between duration of diabetes diagnosis and macrovascular complications is also equivocal. Some studies are in accordance with our findings [37] whereas others have reported the opposite [12, 29]. Similar to the present study, Fox et al. [37] failed to demonstrate duration of diabetes as an independent predictor of combined nonfatal macrovascular events (myocardial infarction, stroke, congestive heart failure, and angina) [37], whereas baseline data from the ADVANCE trial [12] demonstrated that diabetes duration was an independent predictor of nonfatal myocardial infarction and nonfatal stroke among 11,140 individuals with type 2 diabetes aged 55 years and older. Unlike the previous study [12], we were unable to conduct complication-specific analysis due to the small number of reported events.

Consistent with previous research [12, 24–26, 33, 35], longer duration since diabetes diagnosis was independently associated with microvascular complications. Diabetes duration reflects total glycaemic control and risk factor exposure over time [38]. Likewise, both smoking and hypertension have been identified as prominent risk factors in the development of neuropathy, retinopathy, and nephropathy [24–26, 30, 31, 39, 40]. Similar to previous findings [14, 24, 35, 41–43], higher educational attainment was associated with a lower likelihood of microvascular complications in the present study. Education is a universal indicator of SES and is commonly used in cardiovascular epidemiology as it usually remains constant after early childhood and is less likely to be influenced by social changes or illness in adulthood [44]. Lower educational attainment has been associated with poorer disease management, lower rates of physical activity, fewer ophthalmologic visits, and fewer foot examinations [14]. Earlier detection by systematic screening can prevent or delay the development of diabetes-related complications. Reductions in leg amputation rates have been achieved in the UK following changes to the structure of foot care for those with diabetes [45, 46].

In the present study, the risk of microvascular complications was lower in participants who reported a high level of physical activity compared to the lowest physical activity group. This protective effect on microvascular morbidity has been highlighted previously [25]. High levels of physical activity are beneficial for individuals with type 2 diabetes as it is linked with better glucose control [25]. Similar to the present study, the Health and Retirement Study (HRS) in the USA [34] reported that individuals with diabetes-related microvascular complications were less likely to engage in high levels of physical activity. Janevic et al. [34] suggest that the development of diabetes-related complications may cause clinical, practical, and psychological barriers to engaging in physical activity. Therefore, additional support may be needed to achieve the recommended amounts of physical activity in those who have developed complications [34].

The major strength of this study is the large national population-based sample and the high response rate (62%). Inverse probability weights were calculated to take into account the underrepresentation of individuals with lower levels of education attainment and to adjust for the lower response rate in age and sex groupings [20]. Study weights were applied to all analyses to correct for differential nonresponse. Therefore, selection bias was minimised and TILDA sample is representative of the general Irish population [20].

However, several limitations need to be considered when interpreting the findings. Firstly, data used in the analyses were based on self-report and were not ascertained by an objective method. Self-reporting is a recognised limitation in all surveys due to potential inaccuracies and recall and reporting bias [47]. When compared with medical records, data based on self-report have been shown to underestimate the prevalence of diabetic retinopathy [48] and heart failure [49]. In the present study, the prevalence of complications may have been underestimated; as a consequence the measure of association may be biased toward the null. However, moderate-to-high levels of agreement, between self-report and medical records, have been demonstrated for diabetes [49, 50], myocardial infarction [49], stroke [49, 50], and hypertension [49]. Data on smoking and physical activity were also based on self-report where socially desirable responses are a documented phenomenon [47].

Secondly, recall bias should be considered. Participants who were recently diagnosed with diabetes may remember the age of their diagnosis with greater precision. Incorrect reporting may result in differential misclassification and could lead to a subsequent decrease in the measure of association if the number of years since diabetes diagnosis has been overestimated. Nevertheless, the previously documented association between microvascular disease and longer duration since diagnosis [12, 24–26, 33, 35] was detected in this study. Finally, a cross-sectional study design does not permit assessment of causality. For instance, it is not possible to infer if a high level of physical activity reduces the risk of microvascular complications or whether the development of microvascular complications inhibits physical activity [34].

## 5. Conclusions

Despite these limitations, findings from this study are in accordance with other research from prospective cohort studies. We demonstrated that macrovascular complications were more common in the male population and the probability of microvascular complications was reduced in participants with higher educational attainment. Additionally, modifiable risk factors were independently associated with both macro- and microvascular complications. While addressing lifestyle factors is a key part of preventing complications, delivering adequate services for people with diabetes is essential in earlier detection and management of complications. In 2010, a national diabetes programme was introduced in Ireland [51]. To date, the programme has been instrumental in the rollout of a national retinal screening programme, the recruitment of diabetes nurse specialists, and development of a national foot care model [51]. Macrovascular and microvascular complications are often preventable; therefore findings from this study are useful for policy makers planning the development of other diabetes services, including the diabetes cycle of care that has been recently introduced into primary care in Ireland [52]. Diabetes prevalence is projected to increase; therefore effective prevention strategies are urgently needed to reduce the future burden of complications in Ireland.

## Figures and Tables

**Table 1:** Descriptive characteristics of the first-wave TILDA sample aged ≥50 years with diagnosed type 2 diabetes, 2009–2011.

Variable	Type 2 diabetes (total *n* = 655)			
	Male	Female	*p*	Total
	*n* (%)	*n* (%)		% (95% CI)
*Age*				
Years (mean, sd.)	66.6 (9.7)	68.6 (10.4)		67.4 (10.1)
*Location *				
Dublin	92 (25.5)	77 (31.5)	0.17	27.9 (23.1, 33.4)
Another city	113 (30.0)	84 (34.1)		31.7 (26.9, 36.9)
Rural	182 (44.5)	105 (35.3)		40.3 (35.2, 45.7)
*Educational attainment*				
Primary/none	163 (48.6)	116 (54.7)	0.12	51.1 (46.9, 55.4)
Secondary	137 (37.1)	103 (36.1)		36.7 (32.9, 40.7)
Third-level/higher	89 (14.3)	47 (9.2)		12.2 (10.1, 14.6)
*Medical cover*				
State-assisted	163 (43.9)	139 (57.8)	<0.01	49.7 (45.4, 53.9)
Private insurance	111 (27.6)	60 (17.9)		23.6 (20.3, 27.2)
Dual cover	86 (19.9)	51 (18.7)		19.4 (16.2, 23.0)
No additional cover	30 (8.5)	16 (5.6)		7.3 (5.3, 10.1)
*Smoking*				
Never	114 (29.9)	120 (44.0)	<0.001	35.8 (31.8, 39.9)
Past	210 (52.7)	94 (35.4)		45.5 (41.2, 49.9)
Current	66 (17.5)	52 (20.1)		18.8 (15.8, 22.3)
*Physical activity *				
Low	140 (36.8)	143 (56.5)	<0.001	45.0 (40.8, 49.2)
Moderate	137 (35.7)	77 (29.8)		33.3 (29.5, 37.2)
High	109 (27.5)	43 (13.7)		21.8 (18.5, 25.5)
*Age of diagnosis *				
<50 years	54 (16.4)	47 (16.8)	0.04	16.6 (13.7, 19.8)
50–69 years	196 (55.9)	125 (46.2)		51.8 (47.6, 55.9)
70+ years	112 (27.7)	84 (36.9)		31.6 (27.8, 35.7)
*Duration of DM diagnosis*				
Years (median, IQR)	5.0 (1.5, 12)	5.5 (2.5, 15)	0.5	5.3 (2, 11)
*DM treatment*				
*Diet*	64 (16.7)	53 (18.3)	0.9	17.5 (14.8, 20.6)
Oral meds	293 (74.7)	190 (73.9)		74.2 (70.6, 77.5)
Insulin	2 (1.0)	4 (0.8)		0.8 (0.3, 1.8)
Both	31 (8.2)	19 (6.5)		7.5 (5.6, 10.0)
*Other medication *				
Hypertension	221 (58.4)	161 (60.0)	0.6	59.1 (55.4, 63.9)
High cholesterol	164 (43.4)	130 (46.5)	0.4	44.7 (40.4, 48.7)
Flu shot	286 (74.3)	212 (78.5)	0.2	76.1 (72.4, 79.5)
*Doctor diagnosed *				
Hypertension	236 (61.2)	170 (63.8)	0.9	62.5 (58.1, 66.6)
High cholesterol	192 (50.0)	149 (54.2)	0.9	51.7 (47.5, 55.9)
*DM complications*				
Macro	73 (17.8)	28 (11.4)	0.04	15.1 (12.3, 18.4)
Micro	89 (25.2)	67 (26.8)	0.7	26.0 (22.5, 29.9)

**Table 2:** Multivariate binomial regression models exploring independent associations between macrovascular complications and predictor variables^*∗*^.

Predictor	Model 1^†^		Model 2^*ǂ*^		Model 3^§^	
	RR (95% CI)	*p*	RR (95% CI)	*p*	RR (95% CI)	*p*
*Age *						
50–64 years	1		1		1	
65–74 years	1.6 (1.0, 2.5)	0.05	1.5 (0.9, 2.4)	0.07	1.6 (1.1, 2.5)	0.04
75+ years	2.0 (1.3, 3.3)	0.003	1.8 (1.1, 4.1)	0.01	2.0 (1.2, 3.2)	0.005
*Gender*						
Male	1		1		1	
Female	0.6 (04, 0.9)	0.007	0.6 (0.4, 0.9)	0.009	0.6 (0.4, 0.8)	0.005
*Education*						
Primary/less	1		1		1	
Secondary	0.9 (0.6, 1.3)	0.5	0.9 (0.6, 1.4)	0.5	0.9 (0.6, 1.3)	0.6
Third/higher	1.1 (0.7, 1.7)	0.7	1.2 (0.7, 1.8)	0.5	1.1 (0.7, 1.7)	0.7
*Duration of diagnosis*						
0–4 years	1		1		1	
5–9 years	1.0 (0.6, 1.7)	0.9	0.9 (0.6, 1.6)	0.9	1.0 (0.6, 1.7)	0.9
10+ years	1.2 (0.8, 1.8)	0.4	1.1 (0.7, 1.6)	0.7	1.1 (0.8, 1.7)	0.5
*Ever smoked*						
No			1		1	
Yes			1.7 (1.1, 2.7)	0.02	1.6 (1.1, 2.6)	0.04
*Physical activity *						
Low			1		1	
Medium			0.8 (0.6, 1.2)	0.3	0.9 (0.6, 1.2)	0.5
High			0.5 (0.3, 0.9)	0.01	0.5 (0.3, 0.9)	0.03
*Previous hypertension*						
No					1	
Yes					1.1 (0.8, 1.7)	0.5
*Previous high cholesterol*						
No					1	
Yes					1.7 (1.1, 2.5)	0.008

^*∗*^Indicating report of at least one macrovascular complication (heart attack, congestive heart failure, stroke, or TIA).

^†^Variables entered in Model 1: age, sex, education, and years since diagnosis.

^*ǂ*^Variables entered in Model 2: age, sex, education, years since diagnosis, smoking status, and physical activity.

^§^Variables entered in Model 3: age, sex, education, years since diagnosis, smoking status, physical activity, doctor diagnosed hypertension, and doctor diagnosed high cholesterol.

**Table 3:** Multivariate binomial regression models exploring independent associations between microvascular complications and predictor variables^*∗*^.

Predictor	Model 1^†^		Model 2^*ǂ*^		Model 3^§^	
	RR (95% CI)	*p*	RR (95% CI)	*p*	RR (95% CI)	*p*
*Age*						
50–64 years	1		1		1	
65–74 years	0.8 (0.6, 1.2)	0.4	0.9 (0.7, 1.2)	0.4	0.9 (0.7, 1.1)	0.3
75+ years	0.7 (0.5, 1.1)	0.1	0.7 (0.5, 0.9)	0.02	0.6 (0.5, 0.9)	0.01
*Gender*						
Male	1		1		1	
Female	1.0 (0.8, 1.4)	0.9	1.1 (0.7, 1.3)	0.8	1.0 (0.8, 1.3)	0.9
*Education*						
Primary/less	1		1		1	
Secondary	0.9 (0.7, 1.2)	0.4	0.9 (0.7, 1.2)	0.5	0.9 (0.7, 1.2)	0.5
Third/higher	0.6 (0.4, 0.8)	0.007	0.5 (0.4, 0.9)	0.02	0.6 (0.4, 0.9)	0.02
*Duration of diagnosis*						
0–4 years	1		1		1	
5–9 years	1.2 (0.8, 1.8)	0.3	1.1 (0.8, 1.6)	0.5	1.1 (0.8, 1.7)	0.5
10+ years	2.0 (1.5, 2.7)	0.000	1.8 (1.4, 2.5)	0.000	1.9 (1.4, 2.5)	0.000
*Ever smoked*						
No			1		1	
Yes			1.4 (1.1, 2.0)	0.03	1.4 (1.1, 2.0)	0.02
*Physical activity *						
Low			1		1	
Medium			0.7 (0.6, 1.0)	0.1	0.8 (0.6, 1.1)	0.1
High			0.5 (0.3, 0.7)	0.001	0.5 (0.3, 0.8)	0.003
*Hypertension*						
No					1	
Yes					1.5 (1.1, 2.1)	0.006
*High cholesterol*						
No					1	
Yes					1.0 (0.8, 1.3)	0.9

^*∗*^Indicating report of at least one microvascular complication (leg ulcer, protein in urine, neuropathy, retinopathy, or damage to kidneys).

^†^Variables entered in Model 1: age, sex, education, and years since diagnosis.

^*ǂ*^Variables entered in Model 2: age, sex, education, years since diagnosis, smoking status, and physical activity.

^§^Variables entered in Model 3: age, sex, education, years since diagnosis, smoking status, physical activity, doctor diagnosed hypertension, and doctor diagnosed high cholesterol.
